# Atypical visual and somatosensory adaptation in schizophrenia-spectrum disorders

**DOI:** 10.1038/tp.2016.63

**Published:** 2016-05-10

**Authors:** G N Andrade, J S Butler, G A Peters, S Molholm, J J Foxe

**Affiliations:** 1 The Sheryl and Daniel R. Tishman Cognitive Neurophysiology Laboratory Children's Evaluation and Rehabilitation Center, Department of Pediatrics, Albert Einstein College of Medicine and Montefiore Medical Center, Bronx, NY, USA; 2 Departments of Psychology and Biology, The Graduate Center, City University of New York, New York, NY, USA; 3 Trinity Centre for Bioengineering, Trinity College, Dublin, Ireland; 4 Trinity College Institute of Neuroscience, Trinity College, Dublin, Ireland; 5 The Dominick P. Purpura Department of Neuroscience, Rose F. Kennedy Intellectual and Developmental Disabilities Research Center, Albert Einstein College of Medicine, Bronx, NY, USA; 6 The Ernest J. Del Monte Institute for Neuromedicine, Department of Neuroscience, University of Rochester Medical Center, Rochester, NY, USA

## Abstract

Neurophysiological investigations in patients with schizophrenia consistently show early sensory processing deficits in the visual system. Importantly, comparable sensory deficits have also been established in healthy first-degree biological relatives of patients with schizophrenia and in first-episode drug-naive patients. The clear implication is that these measures are endophenotypic, related to the underlying genetic liability for schizophrenia. However, there is significant overlap between patient response distributions and those of healthy individuals without affected first-degree relatives. Here we sought to develop more sensitive measures of sensory dysfunction in this population, with an eye to establishing endophenotypic markers with better predictive capabilities. We used a sensory adaptation paradigm in which electrophysiological responses to basic visual and somatosensory stimuli presented at different rates (ranging from 250 to 2550 ms interstimulus intervals, in blocked presentations) were compared. Our main hypothesis was that adaptation would be substantially diminished in schizophrenia, and that this would be especially prevalent in the visual system. High-density event-related potential recordings showed amplitude reductions in sensory adaptation in patients with schizophrenia (*N*=15 Experiment 1, *N*=12 Experiment 2) compared with age-matched healthy controls (*N*=15 Experiment 1, *N*=12 Experiment 2), and this was seen for both sensory modalities. At the individual participant level, reduced adaptation was more robust for visual compared with somatosensory stimulation. These results point to significant impairments in short-term sensory plasticity across sensory modalities in schizophrenia. These simple-to-execute measures may prove valuable as candidate endophenotypes and will bear follow-up in future work.

## Introduction

Visual processing deficits are widely reported in schizophrenia and hypothesized to have a role in higher-order cognitive and emotional processing deficits.^1, 2, 3, 4, 5, 6, 7^ Research techniques targeting early sensory processing are particularly useful in clinical populations, since they are largely independent of behavioral performance, motivation and attentional state. Studies consistently report decreased amplitudes of early visual evoked potentials (VEP) in schizophrenia-spectrum disorders, particularly of the so-called P1 component occurring 80–120 ms post stimulus.^3, 8, 9, 10, 11, 12, 13^ Although effect sizes are often large in these studies, there is nonetheless substantial overlap in the distributions of amplitudes across patients and controls, limiting clinical applicability of these measures. An obvious research prerogative, therefore, is to establish more sensitive measures of visual sensory dysfunction in schizophrenia to provide greater classification sensitivity. Accordingly, we set out to exploit second-order ‘dynamic' visual processing effects in the form of adaptation of the neural response to repeated stimulations.

The adaptation of neural responses to invariant or repetitive environmental inputs is a fundamental property of sensory processing, and is thought to represent a mechanism by which sensory systems attenuate representational redundancy.^14, 15, 16^ The adaptation studies are abundant in the auditory domain, whereas they are comparatively sparse for the visual sensory modality. Most adaptation studies in the auditory domain use the so-called ‘gating' paradigm, in which robust attenuation in the auditory evoked potential is noted when examining the neural response to the second stimulus in pair as compared with the first (paired presentations^17, 18^). Attenuation of the auditory response can also be elicited by so-called ‘habituation' paradigms, in which the auditory evoked potential attenuation is compared across a block of several stimuli.^19, 20^ A main finding in both auditory habituation and gating studies is that the shorter the period between stimulus presentations, the greater is the attenuation observed.^12, 21, 22, 23, 24^

In the visual adaptation literature, the data are much less clear-cut. Just considering paired-presentation (gating) paradigms, findings are widely varied, encompassing everything from strong visual adaptation to monocular stimulation,^25^ to weakened adaptation to binocularly presented stimuli as compared with other sensory modalities,^26^ to adaptation effects that are specific to right lateral occipital scalp sites,^27^ or no adaptation at all.^28^ It is also interesting to note that the term gating has been applied to paradigms in which non-identical, spatially segregated stimuli are used,^29^ further confusing interpretation of the literature. The effect of presentation rate on adaptation in the visual system has not been widely investigated but consistent with the auditory literature, one study, using blocks of 10 stimuli, reported significantly more gating under ‘fast' as compared with the ‘slow' presentation rates.^30^

Given no clear reliable or independent mechanistic grounding underlying different approaches to studying neural adaptation, the lack of consistency in visual ‘gating' effects, and that there are not many studies using a blocked approach, our group directly compared paired and block visual adaptation paradigms in healthy controls.^31^ Using the exact same stimuli and interstimulus intervals (ISIs), we showed that a blocked paradigm was much more effective than a paired paradigm in driving adaptation, with the data indicating that the visual system required repeated driving of sensory cortex for strong attenuation of the VEP to be observed. This contrasts with auditory processing where single repetitions (paired stimulations) are sufficient to observe short-term sensory plasticity. To avoid propagating the use of inconsistent terminology, we refer to this process as adaptation of the visual response; a label that gets away from the paradigm used to elicit the effect and that may be more representative of underlying mechanisms. In our previous study,^31^ we were able to show that visual adaptation could be observed at the individual participant level, potentially offering a more sensitive measure by which visual processing deficits can be used to characterize those diagnosed with schizophrenia. Here we advance this work by testing the sensitivity of this metric to visual impairment in individuals with schizophrenia.

There is good reason to predict that adaptation to repetitive visual stimuli might be impaired in schizophrenia as patients exhibit other forms of short-term visual plasticity deficits, such as atypical contrast gain control^32, 33^ and motion processing;^34, 35, 36^ as well as the rich literature showing altered adaptation to repetitive auditory stimuli in this population.^28, 37, 38, 39^ This hypothesized reduction in representational redundancy likely serves to enhance the brain's ability to detect more relevant novel environmental changes or novel stimuli. These related processes in which an enhancement of the sensory response to novel or changing stimuli in otherwise uniform sensory environments is expected, indexed for instance by auditory mismatch negativity responses, have also been shown to be impaired in patients with schizophrenia.^40, 41, 42, 43, 44^

In addition, the need for a deeper characterization of visual processing in schizophrenia-spectrum disorders has been highlighted by the ‘Cognitive Neuroscience Treatment Research to Improve Cognition in Schizophrenia (CNTRICS)' consortium^45^ as a domain offering particular promise in novel treatment development.^46, 47, 48^ One way that uncovering visual adaptation deficits may offer hope in this domain is by shedding light on the underlying neurobiology of schizophrenia. For instance, as the visual system is known to be heavily dependent on NMDAR (*N*-methyl-d-aspartate receptor)-mediated activity^49, 50, 51, 52^ and NMDAR activity is thought to be dysfunctional in schizophrenia,^53, 54, 55^ a visual adaptation assay could serve as a proximal, non-invasive read-out informing NMDAR function or change. Further proposed plasticity mechanisms thought to be involved in this and other forms of sensory gating have also been implicated in the pathophysiology of schizophrenia, including both bottom-up and top-down effects relying again on NMDA-mediated glutamate transmission, as well as GABA-ergic inhibition and changes in the ongoing oscillatory activity of the brain (for example, in the gamma band frequency).^5, 56, 57, 58, 59, 60, 61, 62, 63, 64, 65^

Last, as the study of somatosensory adaptation in schizophrenia (SCZ) is largely uncharted territory and has also produced inconsistent findings,^66, 67, 68, 69, 70^ the current investigation also includes a somatosensory analog of our visual paradigm, with an eye to assessing the potential sensory-specificity of short-term plasticity mechanisms. Three studies that we are aware of have paved the way in studying short-term somatosensory adaptation: a 2008 magnetoencephalography study^66^ showing altered secondary somatosensory gating to paired stimuli in schizophrenia; a 2006 electroencephalographic study^70^ showing no somatosensory gating deficits in SCZ, and 2010 magnetoencephalography study^68^ showing altered somatosensory plasticity in a mismatch negativity response task in schizophrenia. For the most part, the more recent work on somatosensation in schizophrenia has focused on graphesthesia and two-point discrimination thresholds.^71, 72, 73, 74, 75, 76^ Although some of these studies have even suggested an endophenotypic role for somatosensory deficits, with for instance, reduced sensitivity in two-point discrimination in both patients and first-degree relatives,^73^ characterization of the basic neurophysiology underlying these phenomena is sparse and warrants further investigation.

## Materials and methods

### Participants

Fifteen adults with a schizophrenia-spectrum disorder (SCZ, four female) and 15 neurotypical adults (NT, five female) completed the visual adaptation experiment. Twelve of the 15 NT and 12 of the 15 SCZ also completed the somatosensory adaptation experiment. All the SCZ participants met DSM-IV criteria for schizophrenia or schizoaffective disorder, using the Structured Clinical Interview for DSM-IV Disorders-Research Version (SCID-R). NT participants had no self-reported history of Axis I or Axis II disorders; Axis I disorders were ruled-out using the Structured Clinical Interview for DSM-IV Disorders-Research Version-NP. Thirteen participants in the SCZ group were receiving antipsychotic treatment (see Table 1). The SCZ group was interviewed using the Positive and Negative Syndrome Scale (PANSS) to quantify current symptom severity. All the interviews were conducted by a certified rater with established research reliability in the administration of these scales. All the participants had normal or corrected vision. All the participants signed informed consent. All the procedures were approved by the Albert Einstein College of Medicine institutional review board and conformed to the tenets of the Declaration of Helsinki. The participants received modest compensation for their participation ($12 per hour).

### Experiment 1. Visual adaptation

#### Stimuli

**Figure 1:**
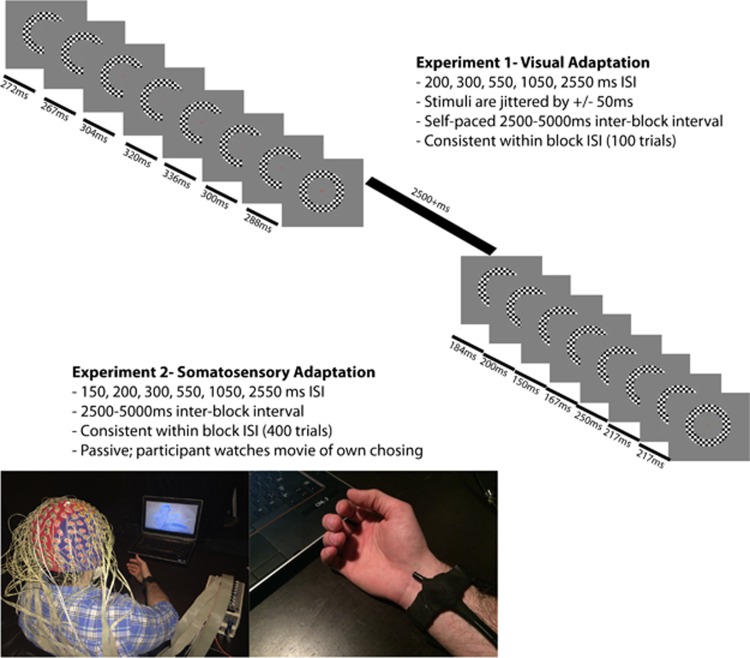
Experimental paradigms. ISI, interstimulus interval.

Stimuli were 100% contrast black and white checkerboard annuli (6.5 cm diameter, 1 cm width, 4^o^ × 4^o^, white luminance of 120 cd m^−2^, black luminance of 0.2 cd m^−2^) centered against a gray (luminance=25 cd m^−2^) background. A fixation cross was always centrally present, including during checkerboard presentation. The cross changed color approximately every 20–40 s, going from red to green for 33 ms and then back to red again. Checkerboards were presented for 33 ms and at different ISIs. Figure 1 displays a schematic representation of the experiment and time course of stimulation.

#### Procedure

The participants sat in a darkened sound-attenuated electrically shielded booth (Industrial Acoustics Company, Bronx, NY, USA), 90 cm from a 34 × 55 cm LCD screen (ViewSonic VP2655wb, 60 Hz refresh). They were instructed to minimize head movements and blinking while fixating on a red cross at the center of the screen. They performed a change detection task to ensure fixation by responding to cross color changes with a button press. The presentation of checkerboards was temporally unrelated to the fixation task.

#### Paradigm

The checkerboards were presented in blocks of 100. Within block, stimuli were centered at an ISI, around which presentations were jittered ±50 ms. Five ISIs were used: 200, 300, 550, 1050 and 2550 ms. Between-block intervals were self-paced; participants initiated the next block by pressing a button 2500–5000 ms after the last stimulus of the preceding block. Block presentation was pseudorandom. In total, the participants experienced four blocks of each of the four shorter ISIs (200, 300, 550, 1050 ms) and two blocks of the longest ISI (2550 ms).^31^ The total run time ranged from 35 to 45 min.

### Experiment 2. Somatosensory adaptation

#### Stimuli

Tactile mechanical stimuli were generated using a custom-built vibrotactile stimulator (Figure 1). The device was worn as a bracelet on the right wrist (see Figure 1), with the stimulator placed over the median nerve.^77, 78^ The device uses a small (4 × 8 mm, 1.1 g) powerful (1.2 G-force, 200 Hz) vibration motor powered by a custom-built 1.5 V amplifier. The stimulus duration was 50 ms, and controlled using Neurobehavioral Systems Presentation Software.

#### Procedure

The participants were seated in a sound-attenuated electrically shielded double-walled booth while a movie of their choice was played on a Dell Latitude E640, at 80 cm viewing distance, with volume adjusted to each participant's personal preference level.

#### Paradigm

The stimuli were presented in blocks of 400. Within the blocks, stimuli were presented at constant ISIs of 150, 200, 300, 550, 1050 or 2550 ms. The ISI block presentation was pseudo-randomized. The participants were exposed to two blocks each of the faster ISIs (150, 200, 300) and one block of the slower ISIs (550, 1050, 2550).

#### Data acquisition (Experiments 1 and 2)

Continuous electroencephalographic data were recorded using a Biosemi ActiveTwo 168 electrode array, analog-to-digital converter and fiberoptic pass-through to a dedicated acquisition computer (digitized at 512 Hz; DC-to-150 Hz pass-band). The data were subsequently low-pass filtered at 45 Hz (fourth-order zero-phase Butterworth filter, 27 dB per octave) and high-pass filtered at 1 Hz (fourth-order zero-phase Butterworth filter, 24 dB per octave). Epochs of 600 ms with 100 ms pre-stimulus baseline were extracted to produce both the VEP and the somatosensory evoked potential (SEP). An automatic artifact rejection criterion of ±75 μV was applied across all the electrodes. Trials with more than eight artifact channels were rejected. In trials with less than eight such channels, bad channels were interpolated using the nearest neighbor spline.^79, 80^ The data were re-referenced to the average of all channels and re-baselined from −100 to 0 ms. For Experiment 1, the adjacent response algorithm was implemented on subject-level data to model and remove response overlap in the fastest ISI condition (150–250 ms).^81, 82^

### Analysis strategy

#### Group-level analysis

**Figure 2:**
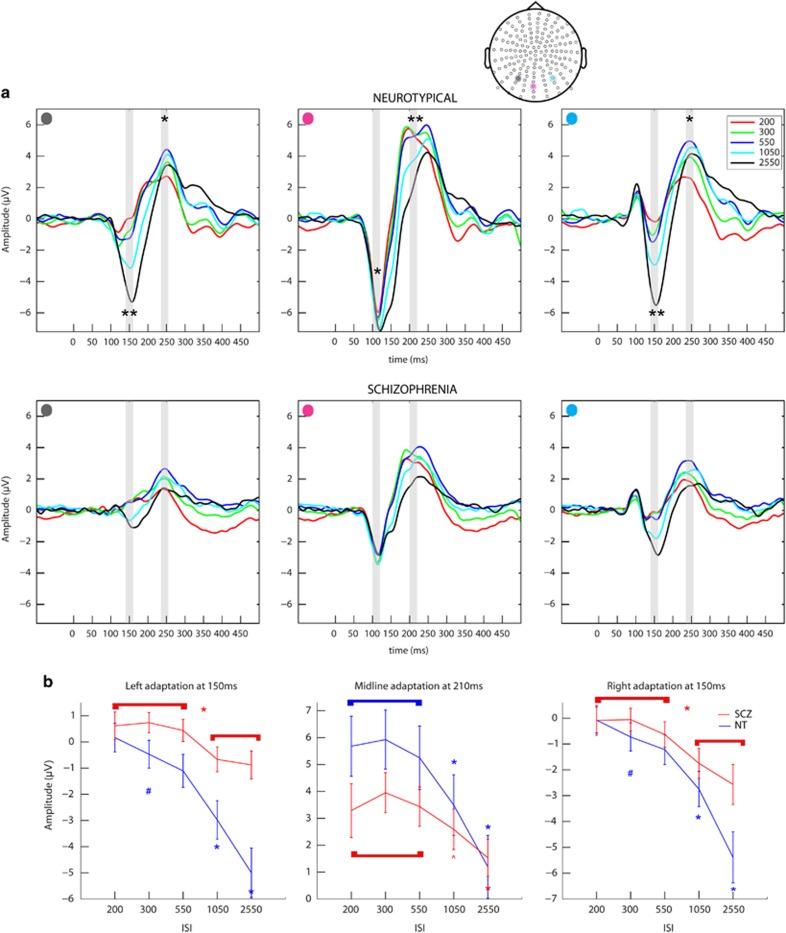
(**a**) Group VEPs—visual evoked responses at the three scalp sites of interest for the neurotypical group (top) and schizophrenia-spectrum group (bottom). Highlighted in gray are the time periods used for statistical analysis. A significant amplitude effect is observed over the midline occipital scalp at an early time period. A significant adaptation effect is observed more laterally at 150 ms and at 200 ms over the midline. (**b**) Tuning curves—representing the significant visual adaptation effects between groups. ISI, interstimulus interval; VEP, visual evoked potential. Top: *, significant main effect; **, significant interaction effect. Bottom: colored *, significantly different from all others; colored ^#^, significant difference between the short ISIs; ^, significantly different from most ISIs; bracket, no significant difference within bracket; if starred, significant difference between brackets.

The analyses were performed using custom MATLAB scripts (Mathworks, Natick, MA, USA), Fieldtrip toolbox for electroencephalography,^83^ EEGLAB^84^ and SPSS (version 20, IBM, Armonk, NY, USA). For both the experiments, the scalp sites and time periods of interest were selected on the basis of maximal activation in the group waveforms and the methods described in the literature.^31, 66, 77, 85^ In brief, group-averaged waveforms were visually inspected across all the scalp sites. This allowed for definition of the precise timing of a given component and delineation of the scalp sites at which each component was of maximal amplitude. For Experiment 1, we restricted analyses to three occipital sites over midline and lateral scalp (Figure 2a) averaged over four time periods of interest: 100–120 ms and 190–210 ms for the midline occipital site, and 145–165 ms and 235–255 ms for the lateral occipital sites. A mixed 2 × 5 repeated-measures analysis of variance (ANOVA), with group as independent factor (SCZ and NT) and ISI as repeated measures (five ISIs), was performed for each scalp site of interest at the appropriate time periods. For Experiment 2, the electrode clusters over midline frontal and left centro-parietal scalp were identified as regions of interest and analyses were performed at a single time period (45–75 ms) surrounding the major prominent SEP peak (Figure 4a). A 2 × 6 mixed repeated-measures ANOVA, with group as the independent factor (SCZ and NT) and ISI as repeated measures (six ISIs) was performed for each region of interest. To provide a more complete picture of the topographic distribution of effects in visual and somatosensory adaptation experiments, scalp potential maps were also generated for time periods of interest.

#### Individual-level analysis

To investigate robustness of adaptation at individual participant level, a non-parametric randomization procedure was conducted.^86^ For each participant, amplitudes recorded under the 2550 ISI condition were compared against each of the other ISI conditions at the scalp sites of interest for the time periods during which adaptation effects differed between groups (for example, significant group × ISI effect in group analysis). The observed difference between the 2550 ms ISI and the test ISI was compared with a reference distribution of differences derived by iteratively randomizing between the two original data sets 10 000 times (that is, individual-subject VEP and SEP amplitudes for the 2550 ms and test ISI). The number of epochs selected for bootstrapping was a subset of the total, which increased in steps of 20 from 30 epochs until statistical significance or the maximum number of sweeps was reached.^87^ A one-tailed threshold of *P*<0.05 defined significance. The *P*-value for a randomization test was calculated from the proportion of values in the reference difference distribution that exceeded the observed difference.^88^ Last, chi-square analysis assessed whether the proportion of participants exhibiting individual-level effects differed between groups.

## Results

### Experiment 1

#### Group-level visual evoked potential analysis

Figure 2a depicts group VEPs for sites of interest for NT (top) and SCZ groups (bottom). Over midline, the first major deflection for both groups was negative-going and peaked at ~110 ms, followed by a second positive going deflection peaking at ~210 ms. Over lateral sites, the first major negative deflection for both groups peaked at ~150 ms, followed by a positive deflection at ~250 ms. Results from the main ANOVA and *post hoc* comparisons for significant group × ISI interactions are presented in Tables 2A and 2B, respectively and summarized below. Figure 2b depicts VEP tuning curves for the time periods during which significant differences in adaptation were noted. As can be seen in the plots, the SCZ group exhibited less ISI-induced modulation, reflected by a more shallow adaptation curve (red), particularly at lateral occipital sites. There was no significant relationship between VEPs and PANSS scores.

### Midline occipital site

*First peak, 100*–*120 ms:* There was a significant main effect of group at ~110 ms over midline occipital scalp, F(1,28)=7.4, *P*=0.01, partial *η*^2^=0.21. Follow-up planned *t*-tests showed significantly reduced VEP amplitudes at all ISIs for the SCZ group relative to NTs (mean group differences ranging from 2.5 to 3.6 μV, all *P*-values <0.04). There was no significant effect of ISI or group × ISI interaction at this time period.

*Second peak, 190*–*210 ms:* A significant ISI by group interaction was observed at ~200 ms over midline occipital scalp, F(4,113)=5.1, *P*=0.006, partial *η*^2^=0.15, indicating that adaptation differed between the groups. To characterize the dynamics of adaptation for the SCZ and NT groups, follow-up within-group ANOVAs and paired *t*-tests were conducted (see Table 2B). These revealed a significant main effect of ISI for each group. At this later time period, the adaptation effect was ‘reversed', with faster ISIs eliciting greater VEP amplitudes, a pattern we previously observed.^31^ Follow-up planned comparisons showed within-group differences in VEP modulation. In the NT group, there were significant differences: (1) when comparing the two slowest ISIs against all other ISIs, and (2) when comparing between the two slowest ISIs, all *P*-values <0.005. In the SCZ group, a similar pattern was observed, all *P*-values <0.05, except the VEP amplitude modulation in comparing the 1050 vs 200 ISI did not reach statistical significance. No significant differences were observed between the shorter ISIs for either group. Figure 2b (middle) provides a summary of these findings.

### Lateral occipital sites

*First peak, 145*–*165 ms:* At ~150 ms, a significant ISI × group interaction was observed over the bilateral occipital sites indicating differential adaptation across groups, left scalp: F(4,112)=7.6, *P*=0.002, partial *η*^2^=0.21; right scalp: F(4,112)=3.8, *P*=0.02, partial *η*^2^=0.12. To unpack how the dynamics of adaptation differed between SCZ and NT, follow-up within-group ANOVAs and paired *t*-tests were conducted (see Table 2B). For the NT group, this revealed a significant main effect of ISI, and the following pattern of significant differences between conditions was observed: (1) when comparing the two slower ISIs against all other ISIs, (2) when comparing within the faster ISIs (550 vs 200) and (3) when comparing between the slowest ISIs (2550 vs 1050), all *P*-values <0.05. In all these comparisons, smaller VEP amplitudes were observed for faster ISIs. For the SCZ group, follow-up comparisons also revealed a significant main effect of ISI. As in the NT group, VEP amplitudes were significantly reduced under the faster ISIs (200, 300, 550) when compared with the slower ISIs (1050 and 2550), all *P*-values <0.05. In contrast to the NT group, in the SCZ group there were (1) no significant VEP amplitude modulations observed when comparing between the faster ISIs (that is, 200, 300 and 550) and (2) no significant VEP amplitude modulations observed when comparing the slowest ISIs (that is, 1050 vs 2550). For both the groups, no significant differences in VEP amplitudes were noted when comparing the two fastest ISIs (200 vs 300). Refer to Figure 2b (left and right) where adaptation curves are plotted. Overall, adaptation mechanisms in the SCZ group are clearly less sensitive, failing to differentiate between the slower stimulation rates as well as between the fastest simulation rates, but showing significant modulation when VEPs recorded to fast vs slow presentation rates were examined.

*Second peak, 235*–*255 ms:* Last, a significant main effect of group was observed over the left occipital scalp at ~250 ms, F(1,28)=5.6, *P*=0.02, partial *η*^2^=0.17, once again reflecting significantly reduced VEP amplitudes in the SCZ group compared with the NT group. A significant main effect of ISI, was also observed over the bilateral occipital scalp at this time, left: F(4,112)=6.4, *P*=0.002, partial *η*^2^=0.19; right: F(4,112)=7.4, *P*=0.001, partial *η*^2^=0.21. The follow-up planned *t*-tests reveal a significant reduction in the VEP amplitude elicited under the fastest ISI (200) as compared with the amplitude under the 300, 550 and 1050 ISI (*P*-values <0.02). Unexpectedly, the VEP amplitude under the 550 ISI was also significantly enhanced compared with all the other ISIs (*P*-values <0.02). There were no significant group × ISI effects noted at this time period over the lateral occipital sites.

**Figure 3:**
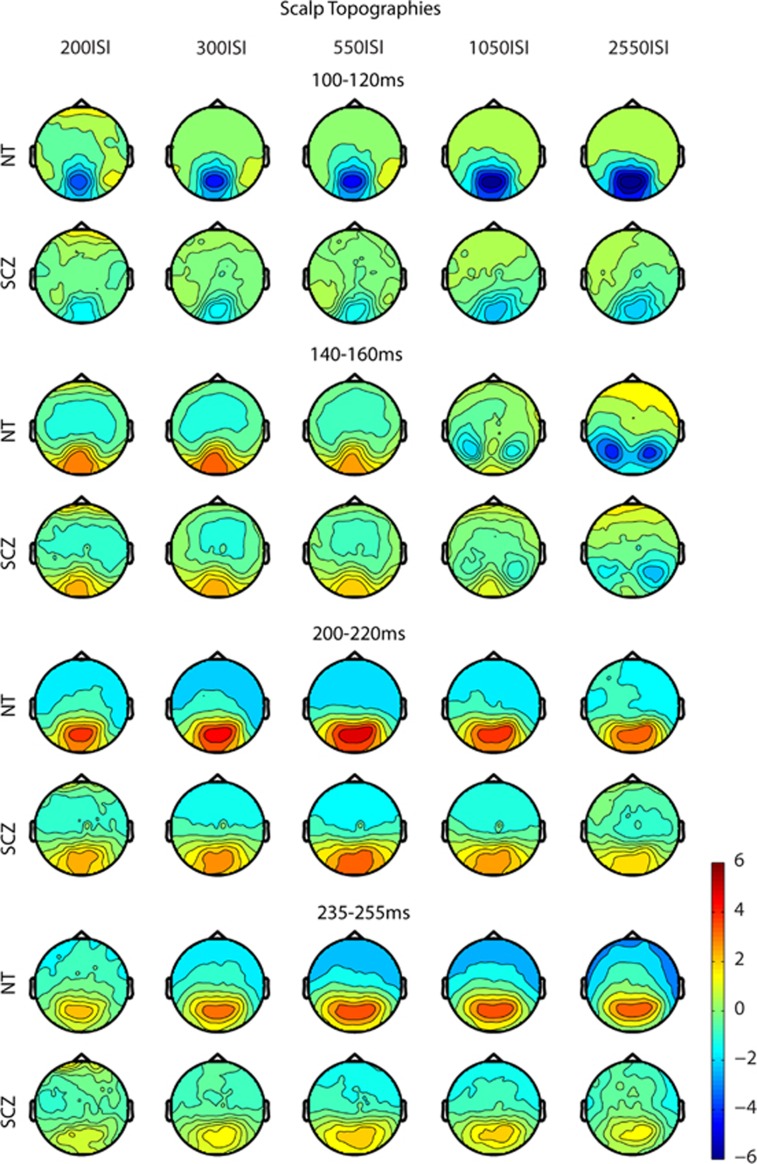
Visual scalp topographic maps—the activity across the entire electrode array is depicted for the five ISI conditions across the time periods used for statistical analysis for both the groups. Overall topography is generally similar between the groups, although amplitude is reduced in the SCZ group. Topographies differ at ~150ms for the two slow ISIs, showing a more bilateral activation pattern as compared with the central occipital pattern noted in the faster ISIs. For the NT group, there is a clear decrease in the amplitude of activation in the topographies with increasingly faster presentation rates (ISIs). ISI, interstimulus interval; NT, neurotypical adult; SCZ, schizophrenia.

Scalp topographic maps for the time periods of interest are presented for each ISI for both the groups in Figure 3, providing a more complete view of the distribution of effects and the evolution of the VEP. Overall the topographies for both the groups are broadly similar (although note the large overall amplitude differences between groups). For both the groups, topographies at ~150 ms for the two slow ISIs showed a more bilateral activation pattern, as compared with the central occipital pattern noted for the faster ISIs. For the NT group, there is a clear decrease in the amplitude of activation in the topographies with increasingly faster ISIs. For the difference in topographies for the time periods in which an adaptation effect between the groups was observed (significant group × ISI interaction), see Supplementary Figure 1.

#### Individual participant-level VEP analysis

Individual-level comparisons were conducted at time periods during which a significant group × ISI effect was observed (see Table 3). Testing at 145–165 ms showed that statistically robust adaptation effects could be established at the individual participant level in nearly all the participants in the NT group when comparing the amplitude to 2550 ms ISI against each of the fast ISIs (200, 300, 550) at both the lateral occipital sites (right: 14 to 15 and left: 13 to 14 participants). When comparing the 2550 ms ISI against the 1050 ms ISI, differences at the individual participant level were seen in 14 NT participants at the right occipital site and 12 at the left. For the SCZ group, when comparing the 2550 ms ISI against the fast ISIs, individual-level differences were noted in 9 to 10 participants at the left occipital site and 10 to 12 at the right; with this number dropping to 7 participants (out of the total 15) when comparing against the 1050 ms ISI at the right occipital scalp. As in the group-level analysis, the single-subject SCZ VEP amplitudes appeared to be less sensitive to modulation elicited by the slower ISI. A chi-square test indicated a significantly greater frequency of adaptors in the 2550 vs 1050 ISI for NT, as compared with the SCZ group for both the lateral occipital sites, left: *χ*^2^(1)=9.6, *P*<0.05; right: *χ*^2^(1)=15, *P*<0.05. There was also a significant difference in the proportion of adaptors in NT when comparing the 2550 vs the fastest ISI for the left occipital site (*P*<0.05) and a trend towards significance for the right occipital site (2550 vs 300, *P*=0.067).

#### Sensitivity of visual adaptation deficits

To further examine the robustness and clinical usefulness of second-order 'dynamic' properties of the visual system, we subjected the VEP measures recorded under a representative 'fast' ISI (300 ms) and the two 'slow' ISIs (1050 and 2550 ms) over the lateral occipital sites (at ~150 ms) to a binary logistic regression. In using these combined adaptation measures to predict group membership (threshold=0.5), we were able to correctly classify 80% of our sample (13/15 SCZ, 11/15 NTs, *P*<0.01). This model performs significantly better than chance and is not improved by the addition of predictors reflecting VEP amplitude differences between the groups (VEP at Oz under the 2550 ISI at ~110 ms).

### Experiment 2

#### Group-level somatosensory evoked potential analysis

**Figure 4:**
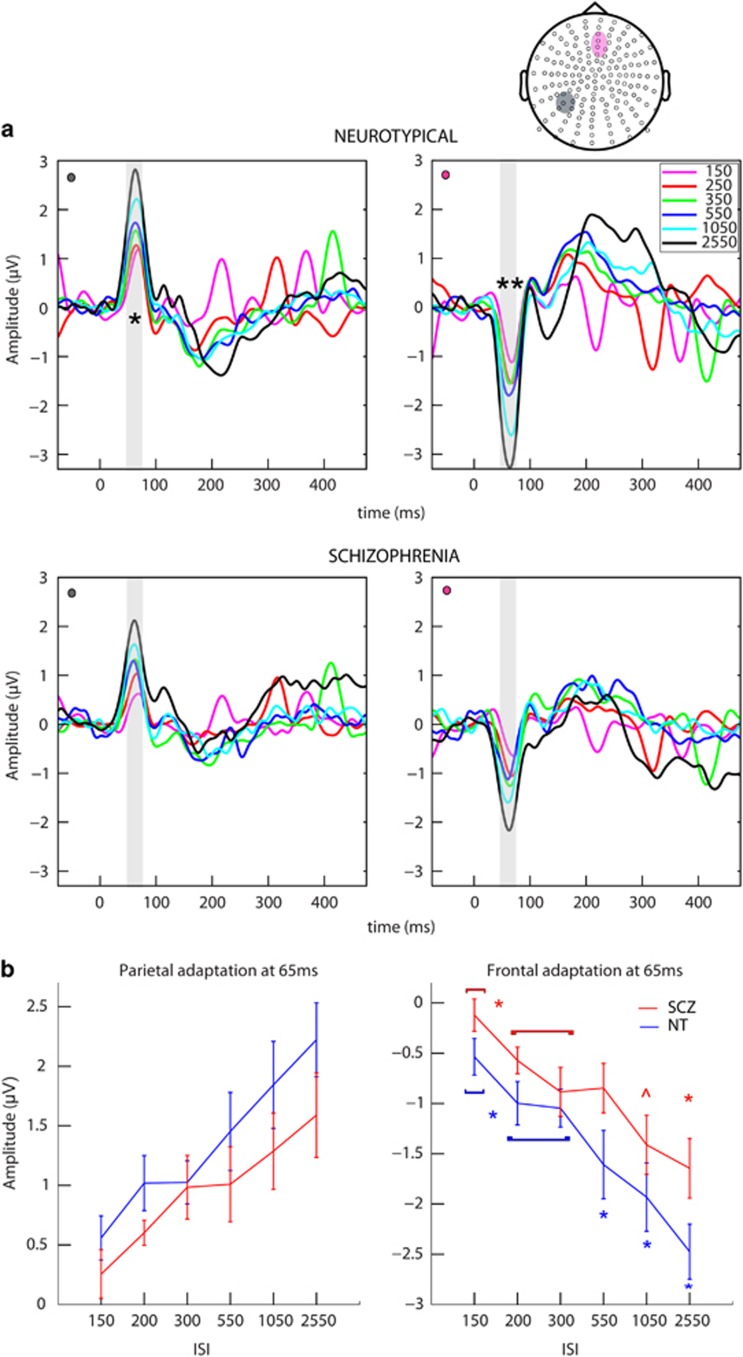
(**a**) Group SEPs—somatosensory evoked responses at the two scalp sites of interest for the neurotypical (NT) group (top) and schizophrenia (SCZ)-spectrum group (bottom). Highlighted in gray is the time period used for statistical analysis. A significant ISI effect but no group differences is observed over the centro-parietal scalp contralateral to stimulation side. A significant adaptation effect is observed over the midline frontal scalp. (**b**) Tuning curves—representing somatosensory adaptation effects in both the groups. ISI, interstimulus interval; SEP, somatosensory evoked potential. Top: *, significant main effect; **, significant interaction effect. Bottom: colored *, significantly different from all others; ^, significantly different from most ISIs; bracket, no significant difference within bracket; if starred, significant difference between brackets.

Both the scalp sites of interest exhibited similar SEP morphology and time course. The response was positive going over the centro-parietal scalp, and inverted over the frontal midline scalp. The SEP amplitude was visibly reduced in SCZ, and as with the visual response this was most apparent for slower presentation rates. Both sites showed a singular prominent peak that inverted across the sites, with maximal amplitude occurring at ~65 ms. Figure 4a depicts the group SEPs for sites of interest for the NT group (top) and the SCZ group (bottom). The results from the main ANOVA and *post hoc* comparisons for significant group × ISI interactions are presented in Tables 4A and 4B, respectively. The main findings are summarized below. Figure 4b depicts the SEP tuning curves for both the groups for both the sites of interest. As can be seen in the plots, the SCZ group exhibited a pattern of ISI-induced modulation that was very similar to that of the NT group, reflected by adaptation curves with quite similar slopes, in contrast to what was observed for the VEPs (Figure 2b).

No significant group or ISI × group effects were observed at the centro-parietal scalp site. However, a significant ISI effect was observed over this site, F(5,110)=24.2, *P*<0.001, partial *η*^2^=0.52. Follow-up planned comparisons revealed a robust adaptation effect, with faster ISIs resulting in significantly reduced SEP amplitudes (all *P*-values <0.03; except when comparing 350 vs 550 ISI, *P*=0.47).

A significant group × ISI effect was observed for the midline frontal site at this time period, F(5,110)=2.9, *P*=0.028, partial *η*^2^=0.12, indicating a difference in SEP adaptation between the groups. To characterize the dynamics of adaptation for the SCZ and NT groups, follow-up ANOVAs and paired *t*-tests were conducted for each group independently. The ANOVAs revealed a significant ISI effect for both the groups, with faster ISIs leading to attenuated SEP amplitudes. Follow-up planned *t*-tests showed robust SEP modulation in the NT group, with significant amplitude differences for all the ISIs pairs, except 200 vs 300. Follow-up comparisons in the SCZ group showed significant differences between the slowest ISI and all other ISIs (2550 vs all), as well as between the fastest ISI and most other ISIs (except 550). Comparisons against and between some of the faster ISIs (300, 200, 550) did not reveal a consistently significant effect. Refer to Figure 4b (right) for the somatosensory tuning curve, a graphical representation of these findings; we present the tuning curve for the centro-parietal site in Figure 4b (left) for comparison. Unlike what was seen in the visual system, somatosensory adaptation differences in SCZ appears less obvious and less consistent. The sensory registration deficits here are also of smaller magnitude and not significant across all the sites examined. There was no significant relationship between SEPs and PANSS scores.

**Figure 5:**
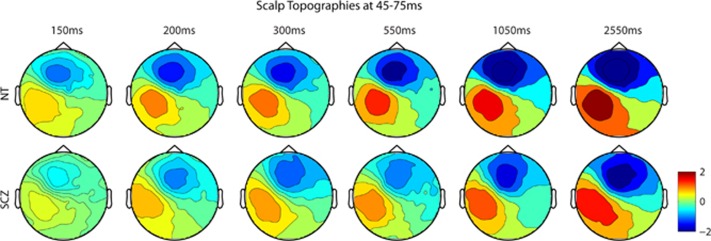
Somatosensory scalp topographic maps—the activity across the entire electrode array is depicted for the six ISI conditions across the time period used for statistical analysis for both the groups. Overall topography is nearly identical between the groups, with a prominent fronto-midline and centro-parietal focus. This topography is consistent between all the ISIs tested. The strength of the activity presented in the topographies decreases as the ISIs become faster. Overall, the amplitude of activation is greater in the NT group. ISI, interstimulus interval; NT, neurotypical adult; SCZ, schizophrenia.

Scalp topographic maps for the time period of interest (45–75 ms) are presented for each ISI for both the groups in Figure 5. Overall, the topographies for the two groups are highly similar, with prominent midline frontal and centro-parietal foci. These are consistent with neural generators in contralateral (to stimulation) somatosensory cortex. This topography is seen across all ISIs, with strength of activity decreasing as a function of faster ISIs. Further, the maps illustrate that the amplitude of the response is diminished for the SCZ compared with NT group, and this is apparent for all ISIs. For the difference in topographies for the adaptation effect between groups (significant group × ISI interaction), see Supplementary Figure 2.

#### Individual participant-level SEP analysis

Individual-level comparisons were conducted for the midline frontal site where a significant group × ISI effect was observed (Table 5). Testing at the 45–75 ms time period revealed significant differences for 11 of the 12 participants in the NT group when comparing the amplitude to 2550 ms ISI against the fastest ISI, with this number dropping to 8 for comparisons against 550, and 5 for comparisons against 1050. For the SCZ group 10 of the 12 participants showed significant differences when comparing the 2550 ms ISI against the 250 ISI, with this number dropping to 4 when comparing against the 550 ms ISI and 3 when comparing against 1050. As in the group-level analysis, there was a drop-off in the sensitivity of SEP amplitude modulation for comparisons between the slower ISIs. However, this drop-off was seen for both the groups. Perhaps a more interesting contrast for this sensory system might be occurring when modulating to one of the next slowest conditions (550 ISI)—as this appears to be the condition in which the number of single-subject adaptors between groups is most discrepant (NT=8, SCZ=4). A chi-square test, however, did not support a significantly greater frequency of adaptors in the NT group compared with the SCZ group for any ISI comparison.

## Discussion

Here, we examined sensory adaptation properties of the visual and somatosensory systems in the participants with a schizophrenia-spectrum disorder using the high-density electrical mapping technique. VEP modulations elicited by parametrically varying stimulation rates were starkly different in patients compared with neurotypical controls, a finding that was most robust over lateral occipital scalp sites. This can be seen in the VEP tuning curves depicted in Figure 2b, where the SCZ group display a considerably shallower adaptation response profile. This less sensitive adaptation was confirmed by formal statistical analyses, with significant differences in VEP amplitude only noted in the SCZ group when the extremes of ISI conditions were compared (that is, the two slower ISIs to the three fast ISIs). No significant differences were noted when comparing between the two slowest ISIs or between any of the fastest three ISIs for the patient group, wholly different to the response profile observed in the NT control group, where clear decrements in response amplitude were observable across almost all increments in the rate of stimulation. In contrast, adaptation profiles in the somatosensory system followed a rather more similar pattern across the groups, as can be seen in Figure 4b where the slopes of the SEP tuning curves for patients and neurotypical controls are largely comparable. On closer examination, significant differences between the groups do arise, but only when very specific comparisons are made (for example, the SEP amplitude elicited under the 1050 ISI is significantly different to responses elicited at all other ISIs for control participants, whereas in patients it is not significantly different for comparisons to the 200 and 300 ISI responses). Differences in basic sensory registration (that is, in the primary sensory response) were also noted in both sensory modalities examined, with patients showing reduced evoked response amplitudes to both visual and somatosensory inputs. As with the adaptation findings, SEP amplitude reductions were substantially less robust than the VEP reductions. The PANSS data were not significantly related to our event-related potential findings. Of note, a previous large-scale study of visual processing in schizophrenia also noted no meaningful association between symptomatology and visual processing deficits in a completely independent and considerably larger sample.^11^ In fact, the small correlation noted between the VEP amplitude and symptom severity scores, was in the opposite direction of what would be expected, with severe symptoms associated with larger VEPs. Further, symptom measures only accounted for 11% of the variance in VEP amplitudes.

Although the current results apparently point to more severe sensory processing issues in the visual than the somatosensory system, it bears emphasizing that the base SEP itself did indeed show significant attenuation in our SCZ cohort, consistent with an early body of work pointing to somatosensory processing dysfunction in this clinical group.^89, 90^ It also bears mentioning that adaptation functions here were only assessed using a single suprathreshold vibrotactile stimulation protocol and that greater deficits might well be uncovered for other somatosensory stimulation types or if stimulation nearer threshold were to be used.

Thus, despite the apparent differences in the severity of adaptation deficits across sensory systems, the presence of basic processing deficits in both sensory systems tested here, and the overwhelming body of evidence pointing to auditory processing deficits,^91, 92, 93, 94, 95^ are all consistent with a 'panmodal' theory of sensory processing deficits in this disorder.^96, 97^ It will be of major research interest to assess whether deficits across sensory systems show similar levels of severity at the individual patient level. It is as yet unknown whether similar underlying deficits at the cellular, synaptic or connectivity level contribute to these deficits across modalities, although this is surely the parsimonious explanation, but it remains possible that the underlying etiology of these deficits could be unique to a given sensory system.

Auditory sensory plasticity, particularly with regard to adaptation and 'gating' of the auditory evoked potential, has been very well characterized in SCZ,^39, 98, 99^ whereas the examination of visual plasticity in this population has been much less frequently examined and has yielded somewhat inconsistent findings.^28, 64, 100, 101^ In turn, considerably more work will be required before somatosensory plasticity in SCZ is fully characterized. In fact, the literature on somatosensory adaptation in healthy controls is still emerging, with one interesting finding pointing to the activation of similar frontal regions in a paired somatosensory gating paradigm and a paired audio gating paradigm in neurotypicals.^102^ This work points to a need in future studies to also examine potential contributions of higher-order regions (that is, outside primary sensory cortices) to sensory gating.

It is of considerable interest that a recent study has reported SEP deficits in a large group of individuals who were determined to be at very high genetic risk for developing schizophrenia,^103^ further suggesting that somatosensory processing deficits may prove useful as endophenotypes for this disorder. Another recent study used the steady-state response technique to assess the inter-trial temporal stability of the cortical somatosensory response in SCZ, with results pointing to both reduced amplitudes and more variable phases of the steady-state response,^104^ highly reminiscent of the early work of Shagass and colleagues.^89, 90^

An obvious question that arises concerns the mechanisms underlying adaptation. In the visual system particularly, we note that the VEP modulation between 'fast' and 'slow' ISIs, while reduced in amplitude, is nonetheless apparent in SCZ and that it is only at finer levels of granularity that deficits become more obvious (for example, in comparing 2550 vs 1050 (slow vs slow) or 200 vs 550 (fast vs fast)). An argument could be made for separate mechanisms underlying adaptation to very fast vs slower sensory stimulation. The fact that both visual and somatosensory systems, in both the groups tested, do not strongly differentiate between each of the fastest ISIs might suggest an adaptive mechanism whereby inconsequential stimulation in rapid succession is simply ‘shut down' to conserve resources. This form of adaptation could result from an active gating mechanism (for example, inhibition) or a passive one, suggestive of a refractory period (for example, depletion). The mechanism underlying adaptation to the slower ISIs may then represent an additional filter. Under these conditions, in NT controls, we observed evoked responses that were still dampened by a faster ISI, but which were also specific to the presentation rate—that is to say that the response to the 1050 ISI was significantly greater than to the 'fast' ISIs, but also significantly smaller than to the next 'slowest' ISI (2550 ms). In SCZ, however, in the visual system, the 1050 ISI did not elicit a response that was significantly different than was seen for the 2550 ISI condition.

In this sense, the event-related potential modulation to the slow ISIs could be said to more closely resemble a 'tuning in' to the presentation rate rather than a 'shutting off' to repetitive stimulation. A recent paper, examining response modulations in the auditory system argues for dissociable effects of so-called 'repetition suppression', elicited by simple repetition, and 'expectation suppression', a different pattern of modulation elicited by the predictability of subsequent stimuli.^105^ In the current study, it is possible that modulations at slower presentation rates rely more heavily on this predictability network, which might be particularly impacted in SCZ. Similarly, separate mechanisms in the auditory system have been recently proposed to explain P50 and N200 adaptation (dubbed 'gating out') and mismatch negativity response and P3 adaptation (dubbed 'gating in').^106, 107^ A related study comparing N1 amplitude across presentation rates in an auditory blocked design also found significant adaptation differences between SCZ and controls at slower ISIs (1 and 4 s) but not at fast ISIs (250 and 500 ms).^108^ The authors there concluded that adaptation to slow and fast stimuli both rely on the same sensory memory systems, suggesting perhaps that no deficits are seen between groups in the shorter ISI in which the auditory memory trace for each stimulus is held only for short periods of time before comparison with the next, as this poses a smaller challenge on the system than longer ISIs. An analogous explanation could also fit the sensory adaptation deficits noted in the current investigation.

Another possible explanation could relate to increased unreliability of neural signals in SCZ when sensory systems are not being driven to depletion. Some have shown that increased unreliability (that is, reduced inter-trial coherence) could account for auditory P50 gating deficits in SCZ^109^ and as mentioned above, this may also account for differences in the somatosensory steady-state response. An unreliable signal could also result from abnormal neural synchrony in local sensory circuits, cortical sensory–frontal circuits and cortical sensory–thalamo–frontal circuits, and aberrant neurotransmitter functioning.^110, 111, 112, 113^ Further, these findings are in line with neurodevelopmental and altered connectivity conceptualizations of SCZ.^114, 115, 116, 117^ Of course, none of these explanations are mutually exclusive and further research will clearly be necessary to disentangle the mechanisms of short-term sensory plasticity observed in the current study and to understand exactly how these measures relate to those obtained in the auditory adaptation experiments discussed above. One might also argue that as amplitude deficits are reported in the literature for patients with schizophrenia, it could be that the VEP in this population simply asymptotes before that of controls, resulting in less room for adaptation effects to be noted, particularly between the responses to the slower ISIs (that is, a ceiling effect). However, examination of individual participant data revealed that even for neurotypical controls with comparable VEP amplitudes (that is, those controls with the lowest amplitude VEPs), adaptation was still evident, whereas in the patient comparators (that is, those patients with the largest VEPs), it was not.

It will also be important to determine whether these adaptation deficits can be useful as endophenotypes of schizophrenia,^118^ a promising proposition given that early visual sensory processing deficits have already been found in healthy first-degree biological relatives of schizophrenia probands.^13^ As an endophenotype, adaptation would lie closer to the ‘shared genetic risk' contributing to the clinical state while being genetically less complex than higher-order symptoms and easier to objectively measure. It will also be informative to assess whether adaptation deficits are related to risk variants on schizophrenia-related risk genes associated with NMDA-mediated processing, because as introduced above, it seems likely that adaptation processes rely heavily on NMDA-mediated mechanisms. Again, it is instructive that basic visual sensory processing differences have already been associated with two NMDA-related genes, *DTNBP1* and *NOS1*, both implicated in schizophrenia risk.^119, 120^ Emerging evidence also implicates NMDA dysfunction in altered somatosensory responses in animal models of schizophrenia.^121, 122^ This convergence of evidence leads us to hypothesize that variation in genes implicated in glutamatergic function may very well influence both visual and somatosensory adaptation.

## Conclusion

In the visual system, robust adaptation deficits, detectable at the individual-subject level, were observed over lateral occipital sites. These electrophysiological markers of visual adaptation were used to correctly classify group membership in 13/15 SCZ and 11/15 NT (80% correct classification rate). To test the specificity of these findings, a somatosensory adaptation experiment was also conducted. Although differences in somatosensory adaptation were noted, the overall SEP adaptation pattern was highly similar between the groups and it is was only in comparing very specific ISI pairs that differences emerged. Decreased VEP and SEP amplitudes were also noted in SCZ. Further study is needed to uncover mechanisms underlying these effects, with altered neuronal synchronization and aberrant glutamatergic signaling being potential candidates.

## Figures and Tables

**Table 1:** Participant characteristics

*Group*	*Age*	*Gender*	*Medication*	*PANSS positive scale*	*PANSS negative scale*	*PANSS general scale*
SCZ (*N*=15)	37 (13)	11 M, 4 F	3 Typical 3 Atypical 7 Atypical+mood 2 None	Mean=19.3, s.d.=6.6, Range: 8–30	Mean=17.3, s.d.=5.8, Range: 9–25	Mean=35.7, s.d.=7.9, Range: 21–49
						
NT (*N*=15)	31 (7)	10 M, 5 F	None			

Abbreviations: NT, neurotypical adult; PANSS, Positive and Negative Syndrome Scale; SCZ, schizophrenia.

The ages are not significantly different; typical/atypical refer to first- and second-generation antipsychotics, respectively; mood refers to antidepressant or mood stabilizer.

**Table 2A:** Visual adaptation—2 × 5 ANOVA

	*Factors*	*Left occipital*	*Midline occipital*	*Right occipital*
1st Peak^a^	Group ISI Group × ISI	F(1,28)=7.4, *P*=0.01 F(4,112)=31.4, *P*<0.001 F(4,112)=7.6, *P*=0.002	F(1,28)=7.4, *P*=0.01 NS NS	NS F(4,112)=33.9, *P*<0.001 F(4,112)=3.8, *P*=0.02

2nd Peak^a^	Group ISI Group × ISI	F(1,28)=5.6, *P*=0.02 F(4,112)=6.4, *P*=0.002 NS	NS F(4,112)=36.4, *P*<0.001 F(4,112)=5.1, *P*=0.006	F(1,28)=3.6, *P*=0.06 F(4,112)=7.4, *P*=0.001 NS

Abbreviations: ANOVA, analysis of variance; ISI, interstimulus interval; NS, not significant; VEP, visual evoked potential.

a Time window for analysis for the first and second most prominent peaks are 100–120 ms and 190–210 ms for the midline VEP, and *t*=145–165 ms and 235–255 ms for the lateral occipital VEPs, respectively.

**Table 2B:** Visual adaptation—follow-up tests unpacking the significant group × ISI effect

*Group*	*Left occipital 1st peak (145*–*165 ms)*	*Midline occipital 2nd peak (190*–*210 ms)*	*Right occipital 1st peak (145*–*165 ms)*
NT	F(4,56)=24.6, *P*<0.001	F(4,56)=24.5, *P*<0.001	F(4,56)=29, *P*<0.001
	2550 vs all, *P*<0.001 1050 vs all, *P*<0.002 550 vs 200, *P*=0.038 300 vs 200, NS	2550 vs all, *P*<0.001 1050 vs all, *P*<0.005 200, 300, 550, NS	2550 vs all, *P*<0.001 1050 vs all, *P*<0.002 550 vs 200, *P*=0.021 300 vs 200, NS

SCZ	F(4,56)=7.5, *P*<0.002	F(4,56)=12.3, *P*<0.001	F(4,56)=8.2, *P*<0.002
	2550 vs 200, 300, 550; *P*<0.03 1050 vs 200, 300, 550; *P*<0.005 200, 300, 550; NS	2550 vs all, *P*<0.02 1050 vs 300, 550; *P*<0.004 200, 300, 550, NS	2550 vs 200, 300, 550; *P*<0.009 1050 vs 200, 300, 550; *P*<0.007 200, 300, 550; NS

Abbreviations: ISI, interstimulus interval; NS, not significant; NT, neurotypical adult; SCZ, schizophrenia.

For each group, the ISI effect is tested with the analysis of variance and planned comparisons (paired *t*-tests) within group are conducted to identify where ISI effect is significant.

**Table 3:** Visual adaptation single-subject analysis

	*2550 vs*			
	*200*	*300*	*550*	*1050*
*Left occipital*				
Neurotypical	15*	14^a^	14^a^	12*
Schizophrenia	9	10	10	9

*Right occipital*				
Neurotypical	13	14^a^	13	14**
Schizophrenia	11	10	12	7

*Midline occipital*				
Neurotypical	7	10	10	9
Schizophrenia	6	10	11	6

a Trend (left: *P*=0.067) in the chi-square analysis, indicating a significant difference in frequency of adaptors between the groups. *P<0.05, ^**^P<0.01.

**Table 4A:** Somatosensory adaptation—2 × 6 ANOVA

*Time*	*Factors*	*Centro-parietal*	*Frontal midline*
45–75 ms	Group	NS	F(1,22)=4.5, *P*=0.046
	ISI	F(5,110)=24.2, *P*<0.001	F(5,110)=50.9, *P*<0.001
	Group × ISI	NS	F(5,110)=2.9, *P*=0.028

Abbreviations: ANOVA, analysis of variance; ISI, interstimulus interval; NS, not significant.

**Table 4B:** Somatosensory adaptation—follow-up tests unpacking the significant group × ISI effect

*Group*	*Frontal midline*
NT	F(5,55)=36.7, *P*<0.001
	2550 vs all, *P*<0.005 1050 vs all, *P*<0.006 550 vs all, *P*<0.04 300 vs 150, *P*=0.002 200 vs 150, *P*=0.013 200 vs 300, NS

SCZ	F(5,55)=16.9, *P*<0.002
	2550 vs all, *P*<0.04 1050 vs 150, 200, 550; *P*<0.05 300 vs 150, *P*<0.002 200 vs 150, *P*=0.01 550 vs 150, 300 vs 200, 550 vs 200, 1050 vs 300, NS

Abbreviations: ISI, interstimulus interval; NS, not significant; NT, neurotypical adult; SCZ, schizophrenia.

For each group, the ISI effect is tested with the analysis of variance and planned comparisons (paired *t*-tests) within group are conducted to identify where ISI effect is significant.

**Table 5:** Somatosensory adaptation—single-subject analysis

*Midline frontal 2550 vs*	*150*	*200*	*300*	*550*	*1050*
Neurotypical	11	10	10	8	5
Schizophrenia	10	8	9	4	3

## Notes

The senior author, JJF, attests that all the authors had access to the full data set and to all stages of the analyses. The authors declare no conflict of interest.
