# Supplementary figures and images for: Atypical visual and somatosensory adaptation in schizophrenia-spectrum disorders
Source: Transl Psychiatry. 2016 May 10;6(5):e804–. doi: 10.1038/tp.2016.63 (PMC5070065; doi:10.1038/tp.2016.63)

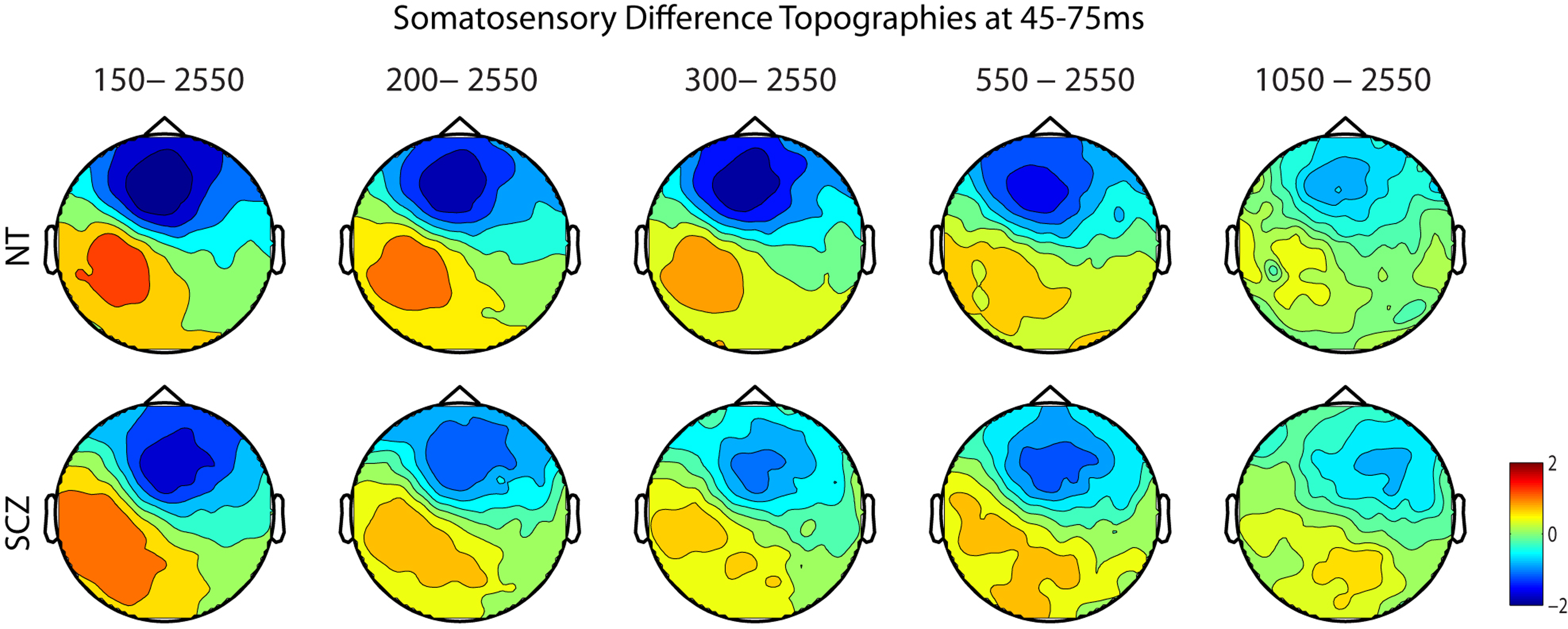

Supplement: Supplementary Figure 2 [file tp201663x2.tif]
