# Supplementary material for: Atypical visual and somatosensory adaptation in schizophrenia-spectrum disorders
Source: Transl Psychiatry. 2016 May 10;6(5):e804–. doi: 10.1038/tp.2016.63 (PMC5070065; doi:10.1038/tp.2016.63)
Supplement: Supplementary Information [file tp201663x3.docx]

**Sup Figure 1. Visual processing difference topographies –** Scalp topographic maps representing the difference between the slowest ISI condition and each subsequent ISI conditions for the time periods in which an adaptation effect between groups (sig Group x ISI) was observed. The difference topographies suggest the local and distal sources might be contributing to the VEP modulation and the differences observed between groups.

**Sup Fig 2. Somatosensory processing difference topographies –** Scalp topographic maps representing the difference between the slowest ISI condition and each subsequent ISI conditions are presented for the time period of interest. The difference topographies suggest a similar pattern of adaptation between groups for the slow vs fast comparisons, with more robust differences noted between ISIs in the NT group.
